# Thyroid, Gonadal and Adrenal Dysfunction in Kidney Transplant Recipients: A Review for the Clinician

**DOI:** 10.3390/biom13060920

**Published:** 2023-05-31

**Authors:** Stefana Catalina Bilha, Simona Hogas, Mihai Hogas, Stefan Marcu, Letitia Leustean, Maria-Christina Ungureanu, Dumitru D. Branisteanu, Cristina Preda

**Affiliations:** 1 Endocrinology Department, “Grigore T. Popa” University of Medicine and Pharmacy, 700115 Iasi, Romania; 2 Nephrology Department, “Grigore T. Popa” University of Medicine and Pharmacy, 700115 Iasi, Romania; 3 Physiology Department, “Grigore T. Popa” University of Medicine and Pharmacy, 700115 Iasi, Romania; 4 Department of Medicine, Charles E. Smith College of Medicine, Florida Atlantic University, Boca Raton, FL 33431, USA

**Keywords:** chronic kidney disease, kidney transplantation, thyroid hormones, sex hormones, cortisol, hypothalamic-pituitary–adrenal axis

## Abstract

While chronic kidney disease-associated mineral and bone disorders (CKD-MBD) prevail in the endocrinological assessment of CKD patients, other endocrine abnormalities are usually overlooked. CKD is associated with significant thyroid, adrenal and gonadal dysfunction, while persistent and de novo endocrinological abnormalities are frequent among kidney transplant recipients (KTR). Low T3 levels prior to transplantation may help identify those at risk for delayed graft function and are often found in KTR. Thyroid surveillance after kidney transplantation should be considered due to structural anomalies that may occur. Despite the rapid recovery of gonadal hormonal secretion after renal transplantation, fertility is not completely restored. Testosterone may improve anemia and general symptoms in KTR with persistent hypogonadism. Female KTR may still experience abnormal uterine bleeding, for which estroprogestative administration may be beneficial. Glucocorticoid administration suppresses the hypothalamic-pituitary–adrenal axis in KTR, leading to metabolic syndrome. Patients should be informed about signs and symptoms of hypoadrenalism that may occur after glucocorticoid withdrawal, prompting adrenal function assessment. Clinicians should be more aware of the endocrine abnormalities experienced by their KTR patients, as these may significantly impact the quality of life. In clinical practice, awareness of the specific endocrine dysfunctions experienced by KTR patients ensures the correct management of these complications in a multidisciplinary team, while avoiding unnecessary treatment.

## 1. Introduction

More than 10% of the general population worldwide suffers from chronic kidney disease (CKD). In 2017, CKD was reported to be the 12th global cause of death (increasing in the past two decades) and accompanied by an ever-growing ranking among the causes of years of life lost [1].

While the cardiovascular, metabolic, hematologic, neurologic and mineral and bone disorders (CKD-MBD) prevail, CKD-associated endocrine abnormalities other than CKD-MBD are usually overlooked. However, CKD is frequently associated with menstrual disorders, sexual dysfunction, infertility and resistance to the effects of growth hormone (GH), all affecting the quality of life [2]. The literature data reports sex-hormone deficiencies, low levels of insulin growth factor 1 (IGF1) and calcitriol, increased prolactin, cortisol and insulin concentrations and thyroid hormone disturbances in CKD [3,4].

Kidney transplantation is generally regarded as the optimal method of renal replacement therapy in most cases of end-stage renal disease [5]. A recent global estimate in 2022 reported a median kidney transplantation prevalence of 255 per million population, with higher accessibility in high-income countries [5]; trends show an annual increase of 2.2% in European countries [6]. While kidney transplantation increases the quality of life, it does not cure CKD-associated metabolic disturbances. Moreover, kidney transplantation also places additional stress upon the body homeostasis due to essential, albeit aggressive, immunosuppressive medication, potentially further impacting the endocrine system [7].

In practice, the interpretation of frequent hormonal perturbations found in samples from CKD and kidney transplant recipient patients (KTR) is challenging for differentiating compensatory from pathological changes, and yet it is crucial for their optimal care.

Hormonal disturbances merit further attention in KTR. Endocrinological investigations in KTR, although readily available, are not routinely performed. Furthermore, result interpretation is cumbersome, as little is known about the specific hormonal changes and the timeline of their occurrence after kidney transplantation compared to the pre-transplantation period.

This manuscript aims to review the main thyroid, sex hormones and cortisol abnormalities in KTR compared to CKD, thus drawing attention to an enhanced partnership between the nephrologist and the endocrinologist in this clinical setting. Many endocrinological abnormalities seen in CKD improve in KTR, but they usually do not disappear completely. Therefore, discussing endocrinological abnormalities in CKD and kidney transplantation together can help clinicians gain a better understanding of the pathophysiology, natural evolution, risks and challenges associated with the management of thyroid, gonadal and adrenal disorders after kidney transplantation.

## 2. Methods

We searched the PubMed electronic database for articles published in the past 20 years (from January 2002 to December 2022) using the keywords “thyroid hormones”/“thyroid”, “sex hormones”/“testosterone”/“estrogen”, “cortisol”/“adrenal” and “kidney transplantation”. Original articles that reported thyroid hormone levels and/or thyroid ultrasound features, sex hormone and cortisol levels in KTR were included. Studies evaluating sex hormones in women included only those referring to premenopausal women for accuracy. Studies reporting data in KTR returning to dialysis or following hormonal substitution therapy were excluded.

In addition, data regarding similar changes in CKD and dialysis patients were also included, if significant. Relevant references from the selected articles were also searched manually.

## 3. Thyroid Hormones and KTR

Thyroid hormones regulate renal structure and hemodynamics, glomerular filtration and water balance [8]. Early animal models showed an association between hypothyroidism and kidney size, structure and weight [9]. Kidney growth retardation in hypothyroid rats occurs mainly due to suppressed cellular and/or nuclear proliferation together with decreased protein synthesis [10]. Reduced kidney mass and a higher prevalence of renal and urologic anomalies are found in children with congenital hypothyroidism. This may be due to shared genes involved in both thyroid and renal organogenesis, such as Pax 8, or due to abnormal embryogenesis related to intrauterine thyroid hormone dysfunction [11]. Thyroid hormones exert both direct and indirect actions upon the kidney function. Hypothyroidism directly decreases sodium and water reabsorption in the renal tubules, leading to filtrate overload that triggers the tubuloglomerular feedback, with preglomerular vasoconstriction and decreased glomerular filtration in experimental models [12]. Indirectly, hypothyroidism leads to impaired myocyte contractility with abnormal systolic and diastolic function. Low concentrations of thyroid hormones are associated with reduced beta-adrenergic response, decreased renin release and renin-angiotensin system (RAS) and reduced atrial natriuretic factor, all negatively impacting renal hemodynamics [13].

While hypothyroidism impacts overall growth in animal models, hyperthyroidism seems to be more selective upon the heart, where it causes cardiac hyperplasia in developing rats [9]. In humans, hyperthyroidism significantly impacts the cardiovascular system, having positive chronotropic and inotropic effects, thus increasing cardiac output and, further on, kidney filtration. Activation of the RAS and increased beta-adrenergic activity contribute to a decrease in afferent arteriolar pressure, with enhanced filtration [13].

On the other hand, CKD patients may experience disturbances in the regulation of the hypothalamic–pituitary–thyroid axis and, thus, kidney dysfunction is frequently associated with alterations in thyroid function tests [14,15]. Advanced CKD is associated with euthyroid sick syndrome, most frequently reflected in low T3 levels. This is due to reduced activity of the peripheral type II-deiodinase due to malnutrition, chronic inflammation, metabolic acidosis, reduced plasma proteins that bind T4 and medication (e.g., exposure to iodinated contrast media, propranolol) [16,17]. Free T3 levels are positively correlated with the estimated glomerular filtration rate (eGFR) in observational studies [9].

The prevalence of subclinical or overt hypothyroidism increases with advancing CKD. CKD stages 4 and 5 were associated with two-fold and three-fold higher odds, respectively, of having thyroid dysfunction in the study of Khatiwada et al. [15]. The thyroid stimulating hormone (TSH) also increases with progressive renal impairment, the prevalence of subclinical hypothyroidism being rather common in stages 4 and 5 CKD: one in four patients with an eGFR < 30 mL/min/1.73 m^2^ in large cohort studies, such as the Third National Health and Nutritional Examination Survey (NHANES III) [18] or more than 400,000 US veterans [19], and up to 43% of stage 5 CKD patients in certain populations [15]. More than half of the cases in NHANES III had subclinical hypothyroidism [18]. Additionally, eGFR < 30 mL/min/1.73 m^2^ was associated with two-fold increased odds of having TSH above the upper limit of normal compared to an eGFR ≥ 90 mL/min/1.73 m^2^ [10]. More so, a 10 mL/min/1.73 m^2^ decrease in the GFR increases the risk of hypothyroidism by 18%, independently of comorbidities [19].

While TSH is considered the most reliable and specific marker for primary hypothyroidism in the general population, this is not necessarily true in CKD patients. Changes in TSH values are common in CKD and do not always reflect thyroid disease, especially in uremic patients. These changes are mainly due to TSH and TRH diminished clearance, abnormal glycosylation of TSH and a reduced response of the pituitary to TRH stimulation. However, TSH is the last thyroid parameter that changes with progressive kidney dysfunction, and it is still more robust when facing non-thyroidal illness [20]. A recent large cohort study among 72,856 individuals did not find a more rapid eGFR decline in subclinical or overt hypothyroidism compared to euthyroid subjects despite lower baseline eGFR in patients with thyroid dysfunction [21]. Renal dysfunction causes thyroid hormone alterations [21], but administering thyroid hormones in patients with kidney dysfunction may even be harmful, as Acker et al. [22] demonstrated in patients with multifactorial acute kidney injury.

Dialysis is also associated with a higher prevalence of hypothyroidism, thyroid nodules and low T3 levels [13]. The prevalence of thyroid dysfunction varies across studies, according to the definition (elevated TSH, subclinical hypothyroidism or overt hypothyroidism). Nonetheless, TSH elevation is reported to occur in 10% to 23% of hemodialysis (HD) or peritoneal dialysis (PD) patients [23]. Free T4 (FT4) concentrations are also reported to be lower in dialysis patients, as a consequence of hormonal removal during dialysis, altered hormonal catabolism, increased iodine stored in the thyroid gland or the presence of thyroid autoantibodies [24].

Low T3 levels were proposed as a predictor of all-cause mortality, but the association was lost after adjusting for hemoglobin, dialysis vintage and history of cardiovascular disease (CVD) in a cohort of 114 HD patients [25]. FT3 levels are also not related to survival in stable HD patients [26]. The low T3 levels are, thus, mirroring the malnutrition and hypercatabolic state seen in severe end-stage renal disease (ESRD) and may represent a physiological adaptation to protein-energy wasting. A prospective cohort of 471 incident HD patients reported an indirect relationship between low T3 levels and all-cause mortality mediated via malnutrition (low normalized protein catabolic rate) and cardiac dysfunction (higher left ventricular mass index and lower ejection fraction) [27].

A large cohort study performed by Rhee et al. [19] also found an increased mortality risk in patients with upper-normal, subclinical and overt hypothyroidism TSH values compared to low–normal TSH in baseline and time-dependent analysis. However, these results from observational studies do not confirm a causal association, as increased TSH values may simply reflect the natural course of the hemodynamic and systemic changes associated with progressive kidney dysfunction [28]. According to Kuczera et al. [3], primary hypothyroidism should be pronounced only when facing elevated TSH levels accompanied by clearly low FT4 concentrations [3]. At the same time, treating an isolated mildly increased TSH (up to 20 mUI/mL in dialysis patients) may promote a negative nitrogen balance and increased muscle catabolism, potentially doing more harm [16].

Low T3 syndrome was found to be persistent after kidney transplantation. In addition, a low pre-transplantation T3 is associated with poor survival of the graft, probably also related to the previous time spent on dialysis [29,30]. Functional thyroid gland disorders are still more common in KTR compared to the general population, despite normalization of the eGFR, and are linked to worse cardiovascular outcomes. Immunosuppressive therapy is certainly a major contributing factor [29].

Schairer et al. [31] found an inverse relationship between dynamic TSH and eGFR during the second-year post-kidney graft receival: each unit (1 µUI/mL) that increased in TSH levels toward the upper limit of normal was associated with a decrease in eGFR of 1.34 mL/min (approximately 2%) within 12 months, after adjustment for age, sex, BMI and comorbidities. The decrease was even more important in patients developing subclinical hypothyroidism (8.7% decrease in 12 months per unit increase in TSH). However, one-time TSH measurements at 12 and 24 months post-transplantation were not related to eGFR changes [31]. Thus, serial measurements of TSH may be more important than one-time determinations.

A positive association was described between serum creatinine and thyroid volume, with the regression of goiter mirroring improving graft function during the early post-transplantation period [32,33]; a negative correlation between free T3 (FT3) and serum creatinine/eGFR was found in older studies, suggesting that FT3 levels actually correlate with the renal graft function on the long-term [30,33]. Low FT3 concentrations, described in up to half of KTR in some studies [34], were also independently associated with endothelial damage and immunosuppressive treatment in the study of Malyszko et al. [35].

On the other hand, administering T3 early in the course of delayed graft function (DGF) for acute tubular necrosis in KTR had no effect on graft function at a one-year follow-up, despite KTR exhibiting typical “euthyroid sick syndrome” [36].

The early drop in TSH, FT3 and FT4 levels [37] (although FT4 remains unchanged according to some authors [38]) reported in the early post-transplantation period probably occurs due to surgical stress, high dose intravenous methylprednisolone or high endogenous catecholamine levels favoring conversion of T4 to reverse T3 rather than to the active T3 [37]; a recovery of the thyroid hormonal system is seen after the third week of the post-transplantation period [37], although TSH and FT3 were still lower when compared to a control group from the general population at six months post-transplantation in some studies [38]. The more significant decrease in FT4 and FT3 in KTR experiencing DGF compared to normal graft function is rather a consequence than a true cause for the DGF and translates into the “euthyroid sick syndrome” due to higher doses of methylprednisolone, more severe reperfusion injury, inflammation and prolongation of the uremic state [37]. Rotondi et al. [39] found that KTR exhibiting pre-transplantation FT3 levels < 3.1 pmol/L had a significantly lower five-year death-censored graft survival rate compared to their counterparts showing pre-transplantation FT3 above the threshold of 3.1 pmol/L. Measurement of pre-transplantation FT3 concentrations might therefore help identify KTR with increased risk for graft failure [39,40].

Immunosuppressive therapy increases the risk of malignancies, including thyroid cancer. An early Polish study identified malignant thyroid nodules (papillary thyroid carcinoma (PTC)) by fine needle aspiration biopsy in 5 out of 44 kidney allograft recipients [41]. A Korean cohort study among 1739 KTR, followed for a mean period of 137 months, reported an incidence of 0.7% (12 cases) of PTC, higher than the usual for the general Korean population in 2008 (0.02%). During a mean follow-up of 94 months for the KTR with PTC, two patients developed locoregional recurrence, while none showed distant metastases. Post-transplantation PTC was associated with a more aggressive lymphatic dissemination [42].

Serum calcitonin is frequently assessed in clinical practice when facing thyroid nodules, especially if suspicious or the patient is young. The kidneys play a significant role in the degradation of calcitonin, and thus calcitonin increases in kidney dysfunction, with levels above 10 pg/mL being common in dialysis patients [43]. Studies demonstrated that KTR also display increased calcitonin levels compared to the general population (9.67 ± 0.66 ug/L versus 6.90 ± 0.43 ug/L, *p* < 0.05 in the study of Sobki et al. [44]), especially in female patients, and this may be due to persistent inflammation or associated infection [44,45]. The main changes in thyroid function are summarized in Table 1.

Key points:

- Low T3 syndrome may persist after kidney transplantation due to corticosteroid administration, inflammation or prolongation of the uremic milieu.
- Pre-transplantation FT3 levels may help identify patients at risk for graft failure.
- Periodical evaluation of thyroid function in KTR is recommended, together with ultrasonographic surveillance, due to chronic immunosuppression and associated risk of malignancy.
- Primary hypothyroidism should be pronounced only when facing elevated TSH levels accompanied by clearly low free T4 concentrations.

## 4. Sex Hormones and KTR

Estrogens exert multiple beneficial renal effects for preventing CKD. Estradiol protects against TGF-β-induced transcription of the type IV collagen gene and also inhibits TGF-β-mediated mesangial cell apoptosis [46,47]. Furthermore, estradiol inhibits type I collagen synthesis in mesangial cells, thus protecting against extracellular matrix accumulation and glomerulosclerosis [48,49]. Estrogens decrease the number of type 1 angiotensin receptors in the kidney and vascular smooth muscle, thus mitigating the sodium-retention and vasoconstriction effects of the RAS system [50]. The estrogen-induced increased expression of type 2 angiotensin receptors is responsible for bradykinin and prostaglandin E2 release, with vasodilatory effects [49,51]. Estradiol also inhibits endothelin expression, possibly via the downregulation of angiotensin II [49].

By contrast, testosterone activates the RAS system, while dihydrotestosterone upregulates the expression of type 1 angiotensin receptors [52,53]. Testosterone is also responsible for podocyte and proximal tubular cell apoptosis in experimental models [54,55]. Male sex also demonstrates increased capillary pressure and hemodynamic stress in response to vasoactive agents compared to women. Finally, men have higher endothelin concentrations compared to women [49,56].

As such, women seem more protected against renal disease progression compared to men due to different glomerular hemodynamics, a direct protective effect of estrogens on cellular processes and, finally, less lifestyle risk factors [49].

A disturbed hypothalamic-pituitary–gonadal axis is frequently encountered in CKD. In men, testosterone deficiency is a common issue in advanced CKD and dialysis. GnRH pulses are hampered in CKD due to uremia and hyperprolactinemia, leading to a loss of the physiological secretion of luteinizing hormone (LH) and secondary hypogonadism. The feedback exerted by testosterone upon the gonadotropin secretion in CKD is also lost due to uremia, resulting in elevated basal LH levels. These elevated levels may, however, include abnormal LH isoforms with less bioactivity due to posttranscriptional changes. Furthermore, Leydig cell resistance to LH action may also be encountered [2,57].

Despite experimental data showing the detrimental effects of testosterone on renal biology, low testosterone concentrations in male CKD patients are paradoxically associated with increased morbidity, mortality risk (mainly due to CVD) and metabolic syndrome, but they are also associated with altered body composition with further loss of muscle mass, osteoporosis and anemia [57]. In a cross-sectional study comparing HD to PD patients, testosterone deficiency (defined as levels < 3 ng/mL, equivalent to <10.4 nmol/L) was more prevalent in HD and correlated with HD technique in multivariate analysis [58]. In another small observational prospective study, serum testosterone was significantly lower in HD compared to non-dialysis CKD, while free testosterone tended to be lower compared to PD patients. A serum testosterone below 2.55 ng/mL was associated with a 3.7% nmol/L higher mortality risk, without reaching statistical significance. The authors propose a free testosterone cut-off value of 54.6 pg/mL for the assessment of survival prognosis [59]. The differences between HD and PD may be explained by less testosterone clearance with PD or greater protein loss with PD, increasing free testosterone [58].

In a larger Canadian cohort study, low testosterone levels (<2.31 ng/mL, equivalent to 7.8 nmol/L) was associated with a higher all-cause mortality in adjusted analysis and with a lower quality of life. The trend toward an association between low testosterone and cardiovascular events was lost after adjusting for age [60]. In the study of Gungor et al. [61], the prevalence of testosterone deficiency, defined as levels below 10 nmol/L, reached 66% in a cohort of 420 HD male patients; each nmol/L increase in serum testosterone decreased the mortality risk by 7%, but the relationship was age-dependent [61]. In another post hoc analysis of ESRD patients (initiating or already undergoing dialysis), 44% of men had testosterone deficiency (<10 nmol/L), 33% had testosterone insufficiency (defined as concentrations between 10 and 14 nmol/L), while 23% had normal testosterone levels (more than 14 nmol/L) [62].

The mechanisms involved in secondary hypogonadism in women are similar to those seen in men. Progesterone deficiency is prominent in the second part of the menstrual cycle, while estradiol levels are usually low, partly due to hyperprolactinemia. The absence of cyclic release of GnRH leads to anovulation [2]. As a consequence, abnormal uterine bleeding is highly prevalent among female CKD patients and the prevalence increases with advancing disease. In dialysis patients, amenorrhea is reported in up to 58% of patients [63]. Premature menopause often occurs [7]. Heavy menstrual bleeding is also reported, and this may worsen any pre-existing anemia and increase the need for erythropoietin stimulating agents. Despite the frequency of menstrual disorders in female CKD patients, only a minority of nephrologists report discussing gynecologic health with their female patients [63].

A recent meta-analysis raises awareness toward a lack of sufficient data regarding the relationship between hypogonadism and cardiovascular risk in female CKD patients, since 12 out of the included 17 studies investigated only male patients. A U-type relationship between estradiol levels and all-cause mortality has been reported in HD, with both low and high levels being associated with a high mortality risk and also with CVD mortality risk in some studies [64].

Sex hormone concentrations are restored in both sexes approximately 4–6 months after a successful kidney transplantation [65,66]. However, the immunosuppressive treatment may still disrupt gonadal homeostasis (Table 2). A sirolimus daily dose is inversely related to testosterone concentrations, even in young men with well-functioning kidneys [67,68]. Circulating serum testosterone is significantly lower in KTR treated with sirolimus compared to those treated with calcineurin inhibitors alone. Different explanations were proposed for these findings, among which were a blocked testosterone synthesis due to impaired gonadotrophin receptor response or even post-receptor abnormalities in the steroidogenesis cascade [69].

Majzoub et al. [72] reported improvement of anemia and hypogonadal symptoms with testosterone therapy in hypogonadal KTR men, without observing any cardiovascular complications [72].

Leydig cell dysfunction is generally rapidly corrected after kidney transplantation, with normal LH concentrations together with prolactin normalization reported three months after kidney transplantation in KTR men treated with tacrolimus, mycophenolate mofetil and steroids [70]. However, Sertoli cell dysfunction is prolonged in KTR, possibly due to glucocorticoid and tacrolimus use [7]. Inhibin B levels significantly decreased after transplantation and remained low [70]. Sperm quality is reported to remain altered up to two years post-transplantation [74]. This may be reflected in the increasing follicle stimulating hormone (FSH) levels reported in KTR [65,70,71]. Despite the hypothesis according to which inhibin B levels are normal or elevated in dialysis patients due to accumulation and decrease with improving renal function [75], the immunosuppressive regimens seem to differently hinder Sertoli cell function: tacrolimus together with mycophenolic acid were associated with decreased inhibin B concentrations in KTR compared to cyclosporin and mycophenolic acid and tacrolimus plus mTOR inhibitor, respectively [65]. 18% of KTR still experience hypogonadism one year post kidney transplantation, especially the patients older than 50. Time on dialysis did not significantly impact the recovery of hypogonadism [76].

Gonadotrophin secretion is also restored in women after successful kidney transplantation, while prolactin returns to the values seen in healthy controls in some [66,73], but not in all, studies [71]. Clinically, improved kidney function is associated with the return of menses. However, the incidence of infertility is higher in female KTR receiving steroids compared to steroid-free regimens [77]. Female KTR are still reported to have lower LH and progesterone levels compared to healthy controls in some studies. Relative hyperestrogenism compared to healthy controls is also reported. Irregular menses and dysfunctional uterine bleeding are reported in female KTR receiving calcineurin inhibitors, although gonadotrophins and prolactin are often within the normal range [59]. The immunosuppressive therapy was thus proposed to affect the ovarian steroidogenic pathway. Hypercortisolism is known to affect fertility via suppression of GnRh pulsatility [78], while experimental data also demonstrate a negative impact upon uterine decidualization [79] and implantation [80]. Although steroids are considered among the safe immunosuppressive regimens during pregnancy, together with azathioprine and calcineurin inhibitors [81,82], they may be, at least partially, responsible for infertility on the long-term in female KTR [7].

In their study, Pietrzak et al. [83] proposed the usage of hormonal contraception in female KTR for regulating menstrual bleeding, preventing the formation of ovarian cysts and ameliorating the patients’ well-being. The transdermal route appears safer. According to the KDIGO Clinical Practice Guideline for the Care of Kidney Transplant Recipients, intrauterine devices may be less effective and are associated with a risk of infection [82].

Key points:

- Restoration of gonadal function takes place soon after successful kidney transplantation.
- Despite testosterone and estradiol concentrations returning toward the normal range, fertility is not completely restored.
- Male KTR still exhibit low sperm quality and low inhibin B levels.
- Female KTR still exhibit abnormal uterine bleeding and anovulatory cycles.
- Steroid immunosuppressive regimens may partially contribute to infertility on the long-term and should be balanced in female KTR planning pregnancy.
- Hormonal contraception, preferably offered by the transdermal route, is beneficial for correcting bleeding abnormalities and improving quality of life in female KTR.
- Testosterone therapy may help improve anemia and hypogonadal symptoms in KTR with low serum testosterone.
- Clinicians should discuss hypogonadism-related health problems with their KTR patients, as immunosuppressive regimens have a detrimental impact upon gonadal health.
- Sirolimus has a worse impact upon gonadal function compared to calcineurin inhibitors.

## 5. The Hypothalamic-Pituitary–Adrenal Axis and KTR

Cortisol levels follow a circadian rhythm and vary significantly throughout the day, with serum concentrations peaking in the morning, declining during the day and reaching a nadir at midnight. The hypothalamic–pituitary–adrenal (HPA) axis is one of the body’s adaptive mechanisms to stress. Morning cortisol may increase in response to various stressors, and thus an increased 8 am plasma cortisol does not always reflect pathological hypercortisolism [84].

Studies reporting morning blood cortisol in CKD patients mainly report the absence of significant changes even in dialysis patients [4]; however, there are some studies reporting a negative correlation between morning cortisol levels and eGFR, with increasing cortisol as renal function declines [4]. In a cohort of hypertensive patients, cortisol levels were negatively associated with the eGFR after adjustment for clinical factors [85]. The presence of comorbidities such as heart failure [4], depressive disorders [86]—which are more frequent in ESRD compared to CKD [87]—or the presence of inflammation [88] may account for the differences among studies. Although cortisol is cleared from the circulation during both HD and PD, blood cortisol levels are not restored. An increased cortisol secretion during dialysis may be involved [4].

Due to the physiology of cortisol secretions, multiple tests are necessary for the screening of true hypercortisolism: late-night cortisol, 24-h urinary free cortisol (UFC) and a low-dose Dexamethasone inhibition test. CKD and ESRD patients have higher evening cortisol levels both in the blood and saliva, suggesting that the decline of cortisol over a 24-h period is attenuated [4,89,90]. Salivary free cortisol is negatively correlated with the eGFR [90].

Despite an increased 24-h exposure to cortisol, UFC does not increase due to reduced renal clearance of cortisol and is not, therefore, a reliable test for hypercortisolism screening in CKD [4,91,92]. The standard 1 mg Dexamethasone overnight suppression test is also less effective in achieving morning cortisol levels < 1.8 µg/dL. This is not necessarily due to the modified pharmacokinetics of Dexamethasone but rather to the prolonged half-life of cortisol subsequent to diminished renal clearance and hindered inactivation via the renal 11β-hydroxysteroid dehydrogenase (11β-HSD), as well as a lower sensitivity of the HPA axis to feedback regulation [4]. Raff et al. [93] also incriminate chronic inflammation and depression as causes of abnormal suppression after low-dose dexamethasone [69]. Cardoso et al. [90] reported normal suppression with the administration of 2 mg Dexamethasone in CKD patients failing to suppress with the standard 1 mg dose.

Adrenocorticotropin (ACTH) blood concentrations also have the tendency to increase in the more advanced stages of CKD, where metabolic acidosis, inflammation and increased stress related to diminished quality of life become apparent [4].

The high dose 8 mg Dexamethasone was proposed to be more accurate when differentiating tumoral hypercortisolism from non-neoplastic hypercortisolism in CKD. This test is usually used to distinguish between pituitary corticotroph adenoma and ectopic ACTH syndrome in the general population. However, the literature data report adequate suppression to less than 1.8 ug/dL for blood cortisol after the 8 mg dexamethasone test in CKD if Cushing disease is absent. Analyzing the circadian rhythm of both cortisol and ACTH performs better in CKD compared to nadir cortisol levels alone [94].

Cushing syndrome, as well as subclinical hypercortisolism, are associated with increased cardiovascular and metabolic risk in the general population [95]. Whether the subclinical hypercortisolism encountered in CKD patients contributes to the increased CVD burden and mortality is still largely unexplored. A pre-dialysis baseline cortisol level > 10 ug/dL was associated with increased adjusted all-cause mortality, higher rates of CVD and left ventricular systolic dysfunction in a recent cohort study of 133 HD patients [96].

On the other hand, diagnosing adrenal insufficiency in dialysis patients is a difficult task, due to falsely elevated cortisol levels. Ohashi et al. [97] proposed the cut-off level of 8.45 ug/dL for morning cortisol as having the highest sensitivity and specificity for predicting adrenal insufficiency in HD patients. Primary adrenal insufficiency should be suspected in HD (1) if the peak serum cortisol is <15 ug/dL in the ACTH stimulation test (according to Ohashi et al. [97]) or under 18 ug/dL (according to Sakao et al. [98]) or (2) if basal plasma ACTH are high without increased serum cortisol in the ACTH stimulation test [97].

Data reporting the function of the HPA axis in KTR are very scarce, probably mainly due to the fact that these patients are exposed to various glucocorticoid immunosuppressive regimens. Long-standing KTR following chronic corticosteroid treatment show a suppressed HPA axis demonstrated by reduced 24 h UFC concentrations that are independently associated with the presence of metabolic syndrome: hypertension, dyslipidemia, central obesity and diabetes [99] (Table 3).

Chronic low-dose prednisolone treatment (5 mg or 7.5 mg prednisolone/day for six months or more) is associated with a suboptimal response to the 250 µg ACTH test in almost half of the KTR that underwent testing in the study of Valentin et al. [103].

Older studies proposed the 1 µg ACTH test in order to test for various degrees of adrenal insufficiency prior to steroid withdrawal [104]: 60% of KTR under steroid treatment for a mean period of 78 weeks had a borderline (peak cortisol to 1 µg ACTH test < 18 µg/dL, but 8 am serum cortisol > 5 µg/dL) or abnormal adrenal reserve (peak cortisol to 1 µg ACTH test < 18 µg/dL and 8 am serum cortisol < 5 µg/dL). The authors did not find any correlation between the duration of steroid treatment and the adrenal test results [104].

In 63 KTR following long-term (mean 36 months) prednisone therapy, one third (31%) tested as suboptimal at the low-dose ACTH stimulation test, while morning cortisol was clearly suppressed in 14% of the patients. Graft function slightly decreased by approximately 5% after step-wise steroid withdrawal and it was more pronounced in symptomatic patients (−7 versus −2 mL, respectively) [100]. This may reflect the physiological effect of eGFR increase exerted by glucocorticoids [105].

Therefore, studies evaluating the adrenal reserve in KTR advocate for endocrinological evaluation before steroid withdrawal and the use of rescue glucocorticoid supplementation during stress or acute illness if borderline results are found [103,104]. Abnormal adrenal reserve (equivalent to overt hypoadrenalism) requires continuation of low-dose physiological steroid replacement treatment. Repeating the stimulation test and 8 am cortisol after three months is also proposed, as significant improvements in adrenal function are found after this timeframe. Baz-Hecht et al. [104] proposed repeating the stimulation test when 8 am cortisol reaches 5 µg/dL or more.

According to the meta-analysis of Broersen et al. [106], there is no steroid treatment dose or duration that can safely exclude adrenal insufficiency, with high doses (above the recommended posology) and long-term administration (>1 year) having the highest risk (21.5% for high dose and 27.4% for long-term, respectively). Medium-term administration (between one month and a year) was associated with an absolute risk of 11.9% of adrenal insufficiency. Nonetheless, adrenal insufficiency was also found even after low-dose (<5 mg prednisolone equivalent dose/day) and short-term administration (<4 weeks) [107]. Current clinical guidelines from the major endocrinology societies—the Endocrine Society and the European Society of Endocrinology—that focus on reducing the risk of glucocorticoid-induced adrenal insufficiency are lacking. General population glucocorticoid withdrawal regimens recommend gradually tapering glucocorticoid doses if administered > three weeks, or administered more than 40 mg prednisolone equivalent/day for more than one week, repeated evening administration of steroids (≥5 mg prednisone equivalent/day in the evening) or a short course of glucocorticoids within one year after long-term steroid administration withdrawal [107,108].

Regarding adrenal reserve testing, recommendations vary: if after withdrawal signs and symptoms occur, and if morning cortisol is between 3 and 15 µg/dL, a short Synacthen test (SST) should be performed [108]; the same cut-offs for dynamic testing in secondary adrenal insufficiency are also endorsed by the Endocrine Society [109]. If cortisol peaks less than 18 µg/dL at ACTH stimulation, physiologic glucocorticoid replacement (15–20 mg Hydrocortisone or 5 mg Prednisone) should be continued and dynamic testing repeated after six months if morning cortisol >12.7 µg or delta cortisol (30′ cortisol–0′ cortisol) at SST > 3.6 µg/dL, or yearly if the values are lower [108]. Other authors recommend measuring early morning serum cortisol 24 h after the last glucocorticoid dose if 5 mg Prednisone or equivalent were administered for more than four weeks prior to withdrawal. If morning cortisol is: (1) above 10 µg/dL, daily glucocorticoids can be stopped without dynamic testing; (2) between 3.6 and 10 µg/dL, continue glucocorticoids with switching to hydrocortisone, and repeat morning cortisol +/− morning ACTH after a few weeks, with dynamic testing if basal measurements are indeterminate; (3) <3.6 µg/dL, continue glucocorticoids with switching to hydrocortisone, and repeat morning cortisol +/− morning ACTH after a few months with dynamic testing if basal measurements are indeterminate [110]. Consensus regarding dynamic testing of the adrenal reserve in KTR is lacking, especially regarding when and whom to test. Functional adrenal testing in KTR reported in the literature was performed after long-term treatment with glucocorticoids (six months to three years), therefore the occurrence of suboptimal adrenal testing is not surprising.

Adrenal insufficiency may occur in KTR despite low-dose prednisolone therapy (5 mg/day): symptomatic (hypotension, hyperpigmentation) adrenal insufficiency due to cytomegalovirus infection was reported five months after renal transplantation in a patient with basal serum cortisol of 4 µg/dL that remained under 10 µg/dL after stimulation with 1 mg tetracosactide. Symptoms improved when the daily dose was increased to 10 mg/day [111].

Therefore, increased alertness regarding the potential occurrence of adrenal insufficiency after steroid withdrawal in KTR is needed. Patients should be informed about the signs and symptoms of hypoadrenalism. Measuring morning serum cortisol either 24 h after the last glucocorticoid dose, or if specific symptoms occur, may be an option for KTR being treated with steroids for more than four weeks prior to withdrawal, as well.

Besides the suppressed HPA axis, the cortisol/cortisone ratio is also reported to be altered in prednisolone-treated KTR. The latter exhibit increased urine cortisol/cortisone ratio, which is inversely related to kidney function, thus suggesting a decreased activity of the type 2 11β-HSD in the kidney. The increased cortisol/cortisone ratio was also linked to an increased mortality risk from infectious causes, independently of the prednisolone daily dose or kidney function [101]. More so, the increase in cortisol/cortisone ratio reported in dialysis patients does not normalize after renal allograft transplantation, suggesting persistent impaired activity of the type 2 11β-HSD [102].

Key points:

- HPA axis suppression usually occurs in KTR due to chronic glucocorticoid treatment.
- The HPA axis may be suppressed even with low-dose glucocorticoid regimens.
- Patients should be informed about the signs and symptoms of hypoadrenalism that may occur after steroid withdrawal.
- Measuring morning serum cortisol either 24 h after the last glucocorticoid dose, or if specific symptoms occur, may be an option for KTR. Performing the SST should be taken into consideration if indeterminate 8 am serum cortisol values (generally accepted cutoffs for SST are between 3 and 15 µg/dL) are found, according to existing data.
- Symptomatic adrenal insufficiency may exceptionally occur in KTR due to various conditions despite low-dose glucocorticoid treatment.
- Chronic glucocorticoid administration increases the risk for metabolic syndrome, while an increased mortality from infection is seen in those with increased cortisol/cortisone ratio.
- A slight reduction in the eGFR may occur after glucocorticoid withdrawal.

## 6. Conclusions

**Figure 1:**
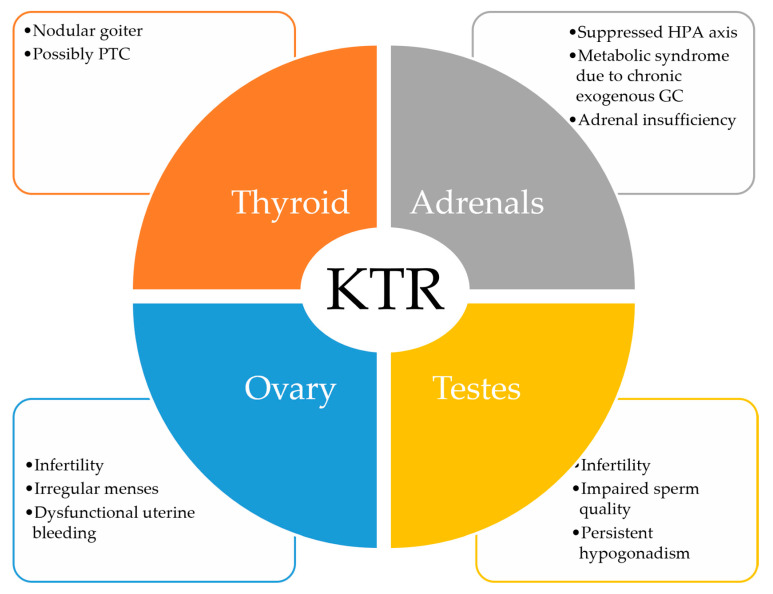
Overall endocrinological abnormalities to clinically assess in KTR. KTR = kidney transplant recipients, PTC = papillary thyroid carcinoma, HPA = hypothalamic–pituitary–adrenal axis, GC = glucocorticoids.

Thyroid, sex hormones and cortisol abnormalities are common in CKD patients, tend to worsen in ESRD and dialysis patients, but are only partially restored after successful kidney transplantation. While the tendency is toward augmented blood levels of the aforementioned hormones, their increase is a mirror of kidney dysfunction rather than a truly pathological process. However, long exposure to these hormonal changes may have a detrimental impact upon the patients’ overall health (Figure 1).

Clinicians should be more aware of the gonadal status of their KTR patients, as women still experience abnormal uterine bleeding after successful transplantation, while refractory or persistent anemia, together with specific hypogonadal symptoms in a male KTR, should prompt testosterone measurement and supplementation if needed. A thyroid ultrasound is advisable after a kidney transplantation, as a higher incidence of thyroid nodules and papillary thyroid carcinoma are reported. Finally, evaluating the adrenal function prior to glucocorticoid withdrawal in KTR is prudent.

## Figures and Tables

**Table 1:** The main changes in thyroid function in advanced CKD versus KTR.

Parameter	Advanced CKD/Dialysis	KTR
Thyroid Hormones		
TSH	↑ [15,18,23]	N/↓ [21,38]
FT4	N/↓ [24]	N [37,38]
FT3	↓ [13,16,17]	↓ [29,30]
Calcitonin	N/↑ [43]	N [44]

CKD = chronic kidney disease, KTR = kidney transplant recipients, TSH = thyroid stimulating hormone, FT4 = free T4, FT3 = free T3, up arrow = increased, down arrow = decreased.

**Table 2:** The main changes in gonadal function in advanced CKD versus KTR.

Parameter	Advanced CKD/Dialysis	KTR
Sex Hormones		
Men		
Testosterone	↓ [2,57,58,59,61,62]	N [67]
LH	↑ [2,65]	N
FSH	↑ [2,65]	↑ [65,70,71]
Inhibin B	N [65]	↓ [65,70]
Women		
Estradiol	↓ [2]	N/↑ [72]
Progesterone	↓ [2]	↓ [72]
LH	↑ [2]	N/↓ [66,73]
FSH	↑ [2]	N/↑ [66,72,73]

CKD = chronic kidney disease, KTR = kidney transplant recipients, LH = luteinizing hormone, FSH-follicle-stimulating hormone, up arrow = increased, down arrow = decreased.

**Table 3:** The main changes in adrenal function in advanced CKD versus KTR.

Parameter	Advanced CKD/Dialysis	KTR
Hypothalamic-Pituitary–Adrenal Axis		
8 am cortisol	N/↑ [4,85] Cut-off: 8.45 µg/dL for predicting AI [97]	↓ * [100]
Late-night salivary cortisol	↑ [90,91]	-
UFC/24 h	↓ [92]	↓ * [101]
Cortisol/cortisone ratio	↑ [102]	↑ [101]

CKD = chronic kidney disease, KTR = kidney transplant recipients, UFC = urine free cortisol. * Due to exogenous glucocorticoid administration. Up arrow = increased, down arrow = decreased.

## Data Availability

No new data were created or analyzed in this study. Data sharing is not applicable to this article.
